# Allele-specific DNA methylation of disease susceptibility genes in Japanese patients with inflammatory bowel disease

**DOI:** 10.1371/journal.pone.0194036

**Published:** 2018-03-16

**Authors:** Hirofumi Chiba, Yoichi Kakuta, Yoshitaka Kinouchi, Yosuke Kawai, Kazuhiro Watanabe, Munenori Nagao, Takeo Naito, Motoyuki Onodera, Rintaro Moroi, Masatake Kuroha, Yoshitake Kanazawa, Tomoya Kimura, Hisashi Shiga, Katsuya Endo, Kenichi Negoro, Masao Nagasaki, Michiaki Unno, Tooru Shimosegawa

**Affiliations:** 1 Division of Gastroenterology, Tohoku University Graduate School of Medicine, Sendai, Japan; 2 Institute for Excellence in Higher Education, Tohoku University, Sendai, Japan; 3 Tohoku Medical Megabank Organization, Tohoku University, Sendai, Japan; 4 Department of Surgery, Tohoku University Graduate School of Medicine, Sendai, Japan; 5 Division of Gastroenterology, Tohoku Medical and Pharmaceutical University, Sendai, Japan; Kurume University School of Medicine, JAPAN

## Abstract

**Background:**

Inflammatory bowel disease (IBD) has an unknown etiology; however, accumulating evidence suggests that IBD is a multifactorial disease influenced by a combination of genetic and environmental factors. The influence of genetic variants on DNA methylation in cis and cis effects on expression have been demonstrated. We hypothesized that IBD susceptibility single-nucleotide polymorphisms (SNPs) regulate susceptibility gene expressions in cis by regulating DNA methylation around SNPs. For this, we determined cis-regulated allele-specific DNA methylation (ASM) around IBD susceptibility genes in CD4+ effector/memory T cells (Tem) in lamina propria mononuclear cells (LPMCs) in patients with IBD and examined the association between the ASM SNP genotype and neighboring susceptibility gene expressions.

**Methods:**

CD4+ effector/memory T cells (Tem) were isolated from LPMCs in 15 Japanese IBD patients (ten Crohn's disease [CD] and five ulcerative colitis [UC] patients). ASM analysis was performed by methylation-sensitive SNP array analysis. We defined ASM as a changing average relative allele score ($\bar{\Delta RAS}$) >0.1 after digestion by methylation-sensitive restriction enzymes. Among SNPs showing $\bar{\Delta RAS}$ >0.1, we extracted the probes located on tag-SNPs of 200 IBD susceptibility loci and around IBD susceptibility genes as candidate ASM SNPs. To validate ASM, bisulfite-pyrosequencing was performed. Transcriptome analysis was examined in 11 IBD patients (seven CD and four UC patients). The relation between rs36221701 genotype and neighboring gene expressions were analyzed.

**Results:**

We extracted six candidate ASM SNPs around IBD susceptibility genes. The top of $\bar{\Delta RAS}$ (0.23) was rs1130368 located on *HLA-DQB1*. ASM around rs36221701 ($\bar{\Delta RAS}$ = 0.14) located near *SMAD3* was validated using bisulfite pyrosequencing. The *SMAD3* expression was significantly associated with the rs36221701 genotype (p = 0.016).

**Conclusions:**

We confirmed the existence of cis-regulated ASM around IBD susceptibility genes and the association between ASM SNP (rs36221701) genotype and *SMAD3* expression, a susceptibility gene for IBD. These results give us supporting evidence that DNA methylation mediates genetic effects on disease susceptibility.

## Introduction

Crohn’s disease (CD) and ulcerative colitis (UC) are the two most common types of inflammatory bowel disease (IBD). IBD is characterized by chronic inflammation of the gastrointestinal tract. The etiology of IBD remains unknown, but accumulating evidence suggests that IBD is a multifactorial disease, influenced by a combination of genetic and environmental factors. Recently, genome-wide association studies (GWAS) have associated more than 200 loci with IBD susceptibility [1–5]. However, these genetic susceptibility loci explain only a small proportion of disease heritability: 13.1% for CD and 8.2% for UC [3]. Most of these 200 loci exist in non-coding regions, with some in gene deserts. Therefore, true IBD susceptibility genes are unconfirmed and the mechanisms governing how IBD susceptibility loci develop the disease are unknown. Conversely, several environmental factors influence disease development and course, including smoking [6, 7], diet [8], and gut microbiota [9, 10]. Genetic and environmental factors influence susceptibility, both independently and interactively. These genome–environment interactions are thought to be mediated by epigenetic modifications of the genome.

Epigenetics is the study of all inheritable and potentially reversible changes in genome function that do not alter the nucleotide sequence within DNA. DNA methylation is the most studied trait in the epigenome. Cytosine methylation occurs on cytosine–guanosine dinucleotides (CpG site), and DNA hyper- or hypomethylation regulates binding of the transcription factor to DNA [11, 12]. Therefore, the status of DNA methylation affects transcription of the gene. DNA methylation appears to be a key in various cellular responses to stimulation from environmental factors. Recently, epigenome-wide methylation association studies (EWAS) have provided insights into other complex diseases such as obesity [13], type 2 diabetes mellitus [14], schizophrenia [15], and rheumatoid arthritis [16]. Moreover, several EWAS of IBD have been reported [17–19]. However, these studies showed different results in hyper- or hypomethylation regions because the samples examined in these EWAS studies were obtained from different tissues and comprised heterogeneous cells; therefore, DNA methylation signatures of IBD remain unconfirmed. Although the number of GWAS and EWAS with IBD is increasing, they have not provided an obvious etiology.

Genetic variants exert an influence on DNA methylation in *cis* [20, 21]; furthermore, these affects *cis-regulatory* to the gene expression [22, 23] and may be a mechanism of genetic–epigenetic interactions in complex disease states. However, there is no evidence for susceptibility single-nucleotide polymorphisms (SNPs) influencing DNA methylation in *cis* in IBD, or supporting evidence for susceptibility allele-specific DNA methylation (ASM) in IBD susceptibility genes.

In this study, we hypothesize that IBD SNPs regulate expressions of the true susceptibility genes by regulating DNA methylation around SNPs. To obtain this supporting evidence, we searched ASM around the susceptibility genes by using methylation-sensitive SNP array (MSNP) analysis [20, 23, 24] and examined if the genotype of the SNP at the ASM site was associated with susceptibility gene expression. DNA methylation profiles may vary among different cell types. Thus, it is important to select appropriate samples, which should include relatively homogeneous, disease-relevant cells [25, 26]. Epidemiological and clinical observations in humans and studies in murine models of IBD suggest that CD4+ T cells are one of the master regulators of intestinal inflammation [27]. Therefore, in this study, we used CD4+ effector memory T cells (Tem) among lamina propria mononuclear cells (LPMCs) isolated from the diseased areas of resected intestines. We selected Tem among CD4+ T cells because the majority of CD4+ T cells in LPMCs were Tem and naïve T cells were very few.

## Materials and methods

### Patient selection and sample collection

Surgical specimens from IBD patients undergoing bowel resection at the Tohoku University Hospital (Sendai, Japan) between July 2015 and September 2016 were used as sources of Tem. Fifteen IBD patients (ten CD and five UC patients) were studied. All patients were Japanese. The demographic features and medications taken by study participants are shown in Table 1. Diagnosis of CD and UC was made based on clinical symptoms and endoscopic, radiographic, and histological findings according to conventional criteria proposed by the Japanese Ministry of Health, Labour and Welfare. The study was approved by the ethics committee and the institutional review board at Tohoku University Hospital. Written informed consent was obtained from all patients prior to participation in this study.

**Table 1:** Clinical characteristics of the patients with Crohn’s disease and ulcerative colitis.

Sample	Age (years)	Sex (M/F)	Sampling site	Disease extent	Medication (active intervention)	Genotype of rs36221701
**CD1**	**40**	**M**	**Colon**	**Ileocolon**	**5ASA, IFX**	**TT**
**CD2**	**26**	**M**	**Ileum**	**Ileocolon**	**5ASA**	**TT**
**CD3**	**29**	**M**	**Ileum**	**Ileum**	**5ASA, IFX**	**TT**
**CD4**	**53**	**M**	**Ileum**	**Ileocolon**	**None**	**CT**
**CD5**	**48**	**F**	**Ileum**	**Ileocolon**	**5ASA, ADA, AZA**	**CT**
**CD6**	**18**	**M**	**Ileum**	**Ileum**	**5ASA, ADA**	**TT**
**CD7**	**35**	**M**	**Ileum**	**Ileocolon**	**5ASA, IFX**	**CC**
**CD8**	**40**	**M**	**Ileum**	**Ileocolon**	**5ASA, IFX**	**TT**
**CD9**	**19**	**M**	**Colon**	**Ileocolon**	**IFX, 6MP**	**CT**
**CD10**	**34**	**M**	**Colon**	**Ileocolon**	**5ASA, ADA, AZA**	**CT**
**UC1**	**59**	**F**	**Colon**	**Pancolitis**	**5ASA**	**TT**
**UC2**	**65**	**F**	**Colon**	**Pancolitis**	**5ASA, PSL**	**TT**
**UC3**	**75**	**F**	**Colon**	**Pancolitis**	**PSL**	**CT**
**UC4**	**65**	**M**	**Colon**	**Pancolitis**	**5ASA, PSL, Tac**	**CT**
**UC5**	**49**	**F**	**Colon**	**Pancolitis**	**5ASA, PSL, AZA**	**TT**

CD, Crohn’s disease; UC, ulcerative colitis; 5ASA, 5 aminosalicylic acid: IFX, infliximab; ADA, adalimumab; AZA, azathioprine; 6MP, 6 mercaptopurine; PSL, prednisolone; Tac, tacrolimus.

### Isolation of LPMCs

LPMCs in the diseased areas of the resected small or large intestines were isolated by the dithiothreitol (DTT)–ethylenediaminetetraacetic acid (EDTA)–collagenase sequences, as previously described [28]. Briefly, the dissected intestinal mucosa was free of mucus and epithelial cells through sequential treatment with DTT and EDTA, and was then digested with collagenase type 3 (Worthington Biochemical Corporation, Lakewood, NJ) and DNase I (Roche, Basel, Switzerland). After collagenase digestion, LPMCs were isolated by density gradient centrifugation with Ficoll–Hypaque (GE Healthcare, Little Chalfont, UK).

### Isolation of Tem in LPMCs and DNA and RNA extraction

CD4+ T cells in the diseased areas of intestines were purified from LPMCs by negative selection using Easy Sep Magnet (STEMCELL Technology, Vancouver, Canada) with Easy Sep Human CD4+ T cell Enrichment (STEMCELL Technology). The isolated CD4+ T cells were purified using FACS aria II cell sorter (BD Biosciences, Franklin Lake, NJ). Cells were stained with anti-CD3-FITC, CD4-PE, CD45RO-APC, CD197 (CCR7)-BV421, and 7ADD-Cell Viability Solution (BD Biosciences), and the Tem were purified. The efficiency of sorting was over 98%. Genomic DNA and total RNA were copurified from isolated Tem using the AllPrep DNA/RNA mini kit (QIAGEN, Hilden, Germany), according to the manufacturer’s instructions.

### ASM analysis (MSNP)

MSNP was performed using the Japonica array according to the method reported by Schalkwyk et al [23]. The Japonica array is an SNP genotyping array designed specifically for Japanese populations [29]. The array contains 659,636 SNPs, including tag-SNPs for imputation, as well as SNPs related to phenotypes from previously reported GWAS and pharmacogenomics studies. We used the Japonica array to analyze patients using three samples: (1) genomic DNA (denoted as G), (2) DNA digested with a cocktail of methylation-sensitive restriction enzymes (MSREs) (denoted as D), and (3) whole-genome-amplified DNA digested with a cocktail of MSREs (denoted as U). Whole-genome-amplification makes DNA fully un-methylated, the (U) samples were used as control samples for the effect of SNPs at enzyme cleavage sites. DNA from patients was digested using a cocktail of three MSREs: HpaII (5′-CˆCGG-3′), HhaI (5′-GCGˆC-3′), and AciI (5′-CˆCGC-3′) (New England BioLabs, Ipswich, USA), which in combination interrogate the methylation status of ~32.4% of CpG sites in the human genome [30]. A cocktail consisting of 1 μL each of three MSREs was diluted by 5 μL CutSmart Buffer (New England BioLabs) and RNase-free water in a total reaction volume of 50 μL, and 1 μg of DNA was digested at 37°C.

The samples of (D) must be DNA from isolated LPMCs because these samples are for analyzing DNA methylation. On the contrary, samples of (G) and (U) are control samples, where (G) are for genotyping and (U) are for determining the effects of SNPs at enzyme cleavage sites; these are not for analyzing DNA methylation. We used DNA from whole blood as the samples of (G) and (U). DNA of (G) and (U) was purified using PAXgene Blood DNA Kit (QIAGEN). Furthermore, we produced unmethylated DNA from patients by using the Illustra GenomiPhi V2 Kit (GE Healthcare), according to the manufacturer’s instructions.

### Analysis of ASM microarray data

**Fig 1:**
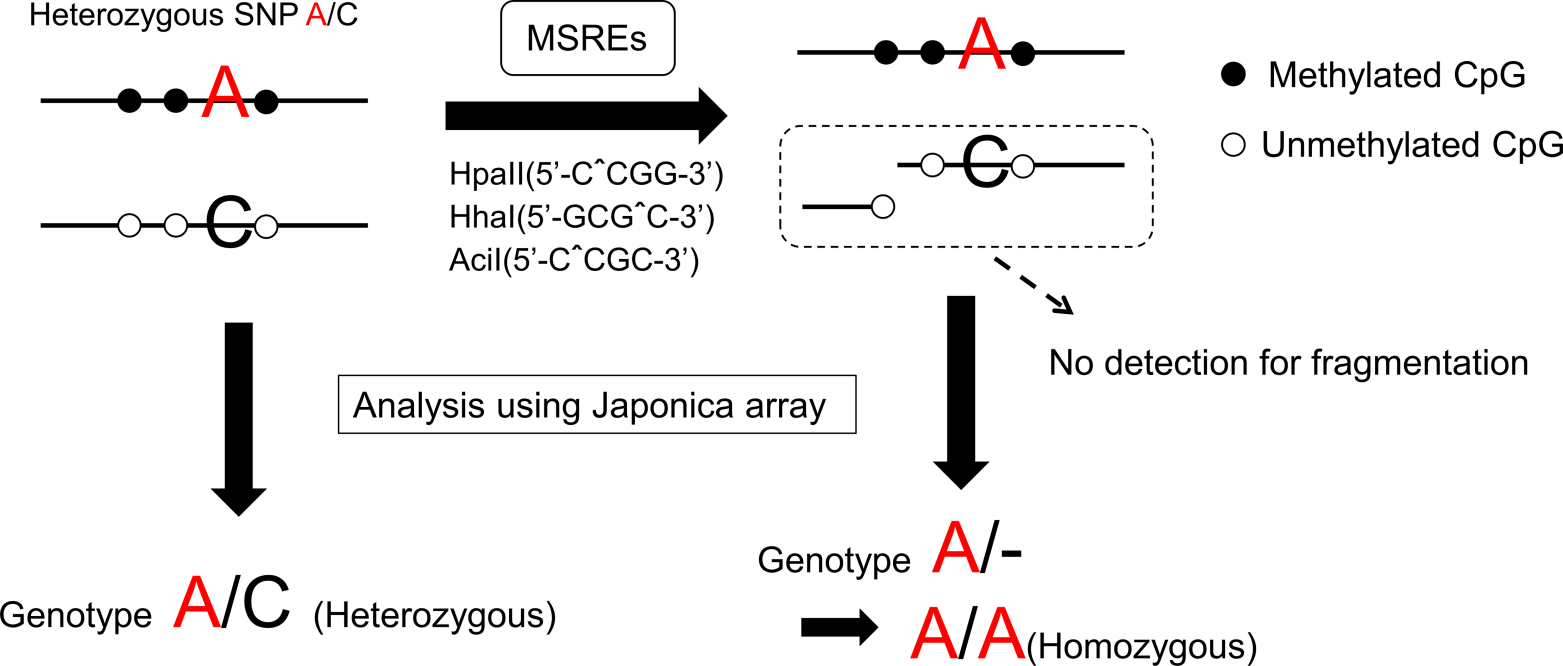
Schema of methylation-sensitive SNP array. If allele-specific DNA methylation (ASM) around heterozygous SNP A/C exists (which is hypermethylated around A allele and hypomethylated around C allele), SNP is called A/C in micro array before digestion and A/A after digestion by MSREs. Thus, the probes heterozygous in uncut genomic DNA and homozygous in MSREs-digested DNA indicate ASM around SNP. Because we expect methylation skew between two alleles, all heterozygous SNPs which the ratio of signal intensities given two alleles changed after digestion should be extracted. SNP, single-nucleotide polymorphisms; MSREs, methylation-sensitive restriction enzymes, which contain HpaII (5′-CˆCGG-3′), HhaI (5′-GCGˆC-3′), and AciI (5′-CˆCGC-3′).

The Japonica array has probes of 659,636 SNPs; among these, the analysis population comprises probes that have sites cut by a cocktail of three MSREs (HpaII, HhaI, and AciI). In genotyping, the main procedures of quality control (QC) usually exclude SNPs or samples with low call rates or biased genotype frequencies. However, the DNA samples in this study were fractionalized by MSREs at many sites and the call rates were necessarily lower. Consequently, we could not follow the standard QC procedures while using the Japonica Array. To inform MSNP, SNP must be heterozygous. The allele call conversion, AB heterozygous in uncut genomic DNA (G) changing to AA or BB homozygous in digested DNA (D) after MSREs digestion, indicate having typical ASM around SNPs (Fig 1). However, the ASM may not occur as a complete (100%) methylation of one specific allele; a limited part of the allele could be methylated. In these cases, the allele call conversion will not occur as there is no call in the digested samples. To detect all of the possible ASM regions, we scaled the raw intensities of each probe set and valued the relative allele score (RAS) [31] to compare the ratio of signal intensities. RAS was defined as A/ (A + B), where A and B are the intensities of the probes for two alleles at a given SNP. We used the Axiom Analysis Suite (Version 2.0.0.35, Affymetrix, Santa Clara, USA) to analyze and view the signal intensities of array data.

For a given SNP in a heterozygous individual, a difference in RAS between (G) and (D) arrays is indicative of ASM. We defined ASM as a change in the average RAS ($\bar{\Delta RAS}$) >0.1. This threshold was established according to a previous report [23]. Two exclusion criteria are shown below.

Exclusion criterion:

$$distance\;(\bar{G})/distance\;(\bar{U})<1.2\;(=\sqrt{\left({\bar{A_{G}}}^{2}+{\bar{B_{G}}}^{2}\right)}/\sqrt{\left({\bar{A_{U}}}^{2}+{\bar{B_{U}}}^{2}\right)}<1.2)$$ (1)

Exclusion criterion:

$$distance\;(\bar{D})/distance\;(\bar{U})<1.2\;(=\sqrt{\left({\bar{A_{D}}}^{2}+{\bar{B_{D}}}^{2}\right)}/\sqrt{\left({\bar{A_{U}}}^{2}+{\bar{B_{U}}}^{2}\right)}<1.2)$$ (2)

A_G_, A_U_, and A_D_; signal intensities of A allele of (G), (U), and (D).

B_G_, B_U_, and B_D_; signal intensities of B allele of (G), (U), and (D).

Exclusion criterion (1) was defined to exclude the probes that did not demonstrate decreasing intensities of (U) compared with (G) and that were not cut accurately by MSREs, or cut accurately but hybridized and detected the intensities. Exclusion criterion (2) was defined to exclude the probes where decreasing intensities of (D) were the same as (U) and those that had unmethylated CpG in both alleles and were cut by MSREs.

**Fig 2:**
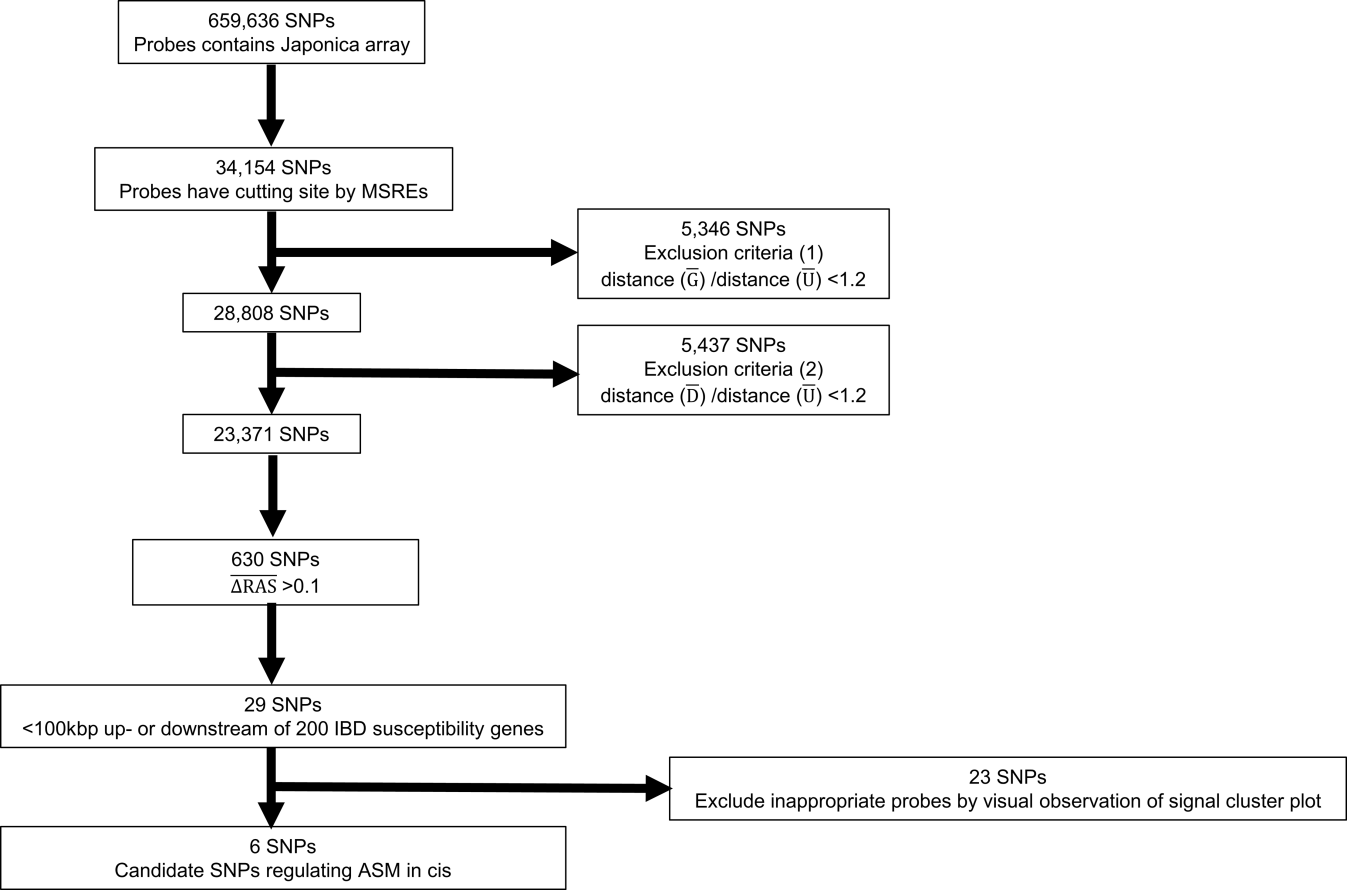
Flowchart of *cis*-regulating allele-specific DNA methylation SNP candidate selection around IBD susceptibility genes. SNP, single-nucleotide polymorphism; MSRE, methylation-sensitive restriction enzyme; RAS, relative allele score; IBD, inflammatory bowel disease; (G), genomic DNA; (D), DNA digested MSREs; (U), whole-genome-amplified fully unmethylated DNA digested MSREs.

**Fig 3:**
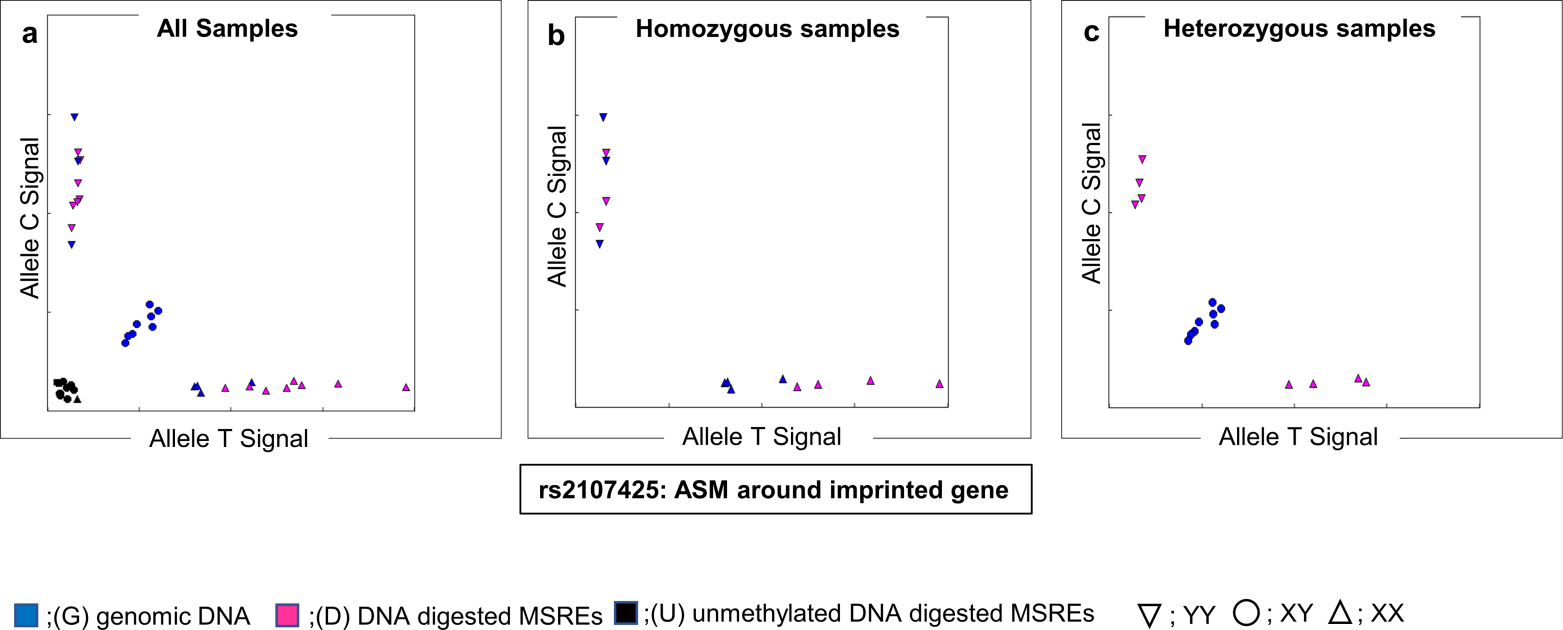
The signal cluster plot in the genomic imprinting region by methylation-sensitive SNP array (MSNP) analysis. (a, b, c) Array data of rs2107425 located on imprinting control region (ICR) showing ASM regulated dependent on paternal or maternal allele. Rs2107425 is located upstream of *H19* (11p15.5), this region is known ICR of *H19* and *IGF2*, which are one of the famous imprinted genes. (a) All samples, (b) Homozygous samples, (c) Heterozygous samples. Heterozygous changed homozygous after digestion. This change was biallelic (C/T changed C/C or T/T); thus, ASM regulated not in *cis* with genotype, but rather due to genomic imprinting.

Among the probes where $\bar{\Delta RAS}$ >0.1 after digestion, we extracted those located on tag-SNPs of 200 IBD susceptibility loci, within 100 kbp up- or downstream of susceptibility genes for IBD around the tag-SNPs, as reported by the International Inflammatory Bowel Disease Genetics Consortium (IIBDGC) [1, 5, 32]. Finally, candidate SNPs around IBD susceptibility genes were extracted, excluding inappropriate probes by visual observation of the signal cluster plot (Fig 2). Genomic imprinting is a good example of ASM. Fig 3 shows the signal cluster plot of rs2107425 located around the well-known genomic imprinting region. This SNP is located upstream of *H19* gene (11p15.5), and this region is a known imprinting control region (ICR) of *H19* and insulin-like growth factor 2 (*IGF2*). *H19/IGF2* ICR is unmethylated in the maternal allele, while methylated in the paternal allele [11, 33]. Thus, the existence of ASM might depend on maternal or paternal alleles around this SNP. The allele call conversion is shown in Fig 3C. All of the C/T heterozygous call in genomic DNA changed into the homozygous call after digestion by MSREs. However, the changed calls were not only one-specific allele homozygous; both CC and TT were observed. This is the difference between the ASM caused by imprinting and SNP. Because our objective was to find the ASM caused by SNP, we needed to find the SNPs that the allele call, after digestion by MSRE, moves in one direction.

The accession number for the genotyping data deposition at NCBI Gene Expression Omnibus repository is GSE110534.

### ASM analysis (bisulfite pyrosequencing)

**Fig 4:**
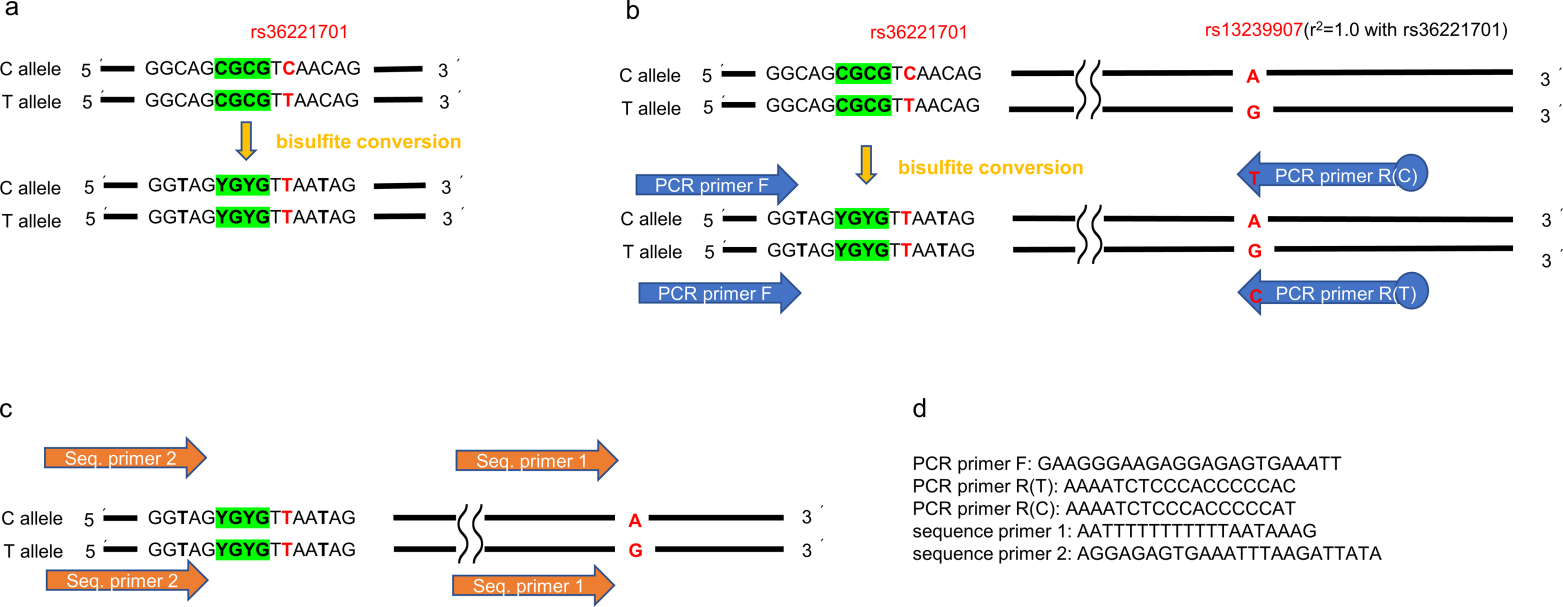
Allele-specific bisulfite pyrosequencing. To analyze the allele-specific DNA methylation, we individually measured the DNA methylation of each allele and compared methylation between the two alleles. Thus, the allele-specific polymerase chain reaction (PCR) was performed and the methylation levels of each of the two alleles, given allele-specific amplicons, were measured separately using pyrosequencing. The allele-specific PCR primers were designed such that each primer of 3′ end was a pair of base of heterozygous SNP. (a) The reference sequence of rs36221701 before and after bisulfite conversion. Rs36221701 contained two CpG sites denoted as CpG1 and CpG2 in 5′ to 3′ direction. By bisulfite conversion, unmethylated C is converted to U (U is converted to T by PCR). Rs362210701 (C/T) is converted to (T/T); it was impossible to distinguish the converted T and original T. Thus, we were unable to detect the origin of the allele for this SNP by PCR primers. (b) Allele-specific PCR. We designed PCR primer for rs13239907 (A/G), which is located 297 bp downstream of rs36221701 and was in complete linkage disequilibrium with rs36221701 (A→C, G→T: r^2^ = 1.0) in JPT data from 1,000 genome project. The forward primer was common to both alleles; reverse primers were designed such that each primer of 3′ end was a pair of bases of genotypes (A or G: complementary strand). (c) Allele-specific pyrosequencing. The two given allele-specific amplicons were separately analyzed using pyrosequencing. Sequence primer 1 targeted rs13239907 and was used to check the accuracy of allele-specific PCR, whereas sequence primer 2 targeted CpG1 and CpG2 was used to analyze the ratio of methylation around rs36221701. (d) Primer details. The 3′ end of PCR primer R was a pair of base of heterozygous SNP. 5′ end of PCR primer R was biotinylated.

To validate results from microarray analyses, we tested ASM (rs36221701) detected by MSNP using bisulfite pyrosequencing. The probe set of rs36221701 contains two CpG sites, which we denoted as CpG1 and CpG2 in the 5′ to 3′ direction. We analyzed genomic DNA samples extracted from Tem in the intestinal tissues of four individuals with heterozygous (C/T), three individuals with homozygous (T/T), and one individual with homozygous (C/C) at the locus. To analyze ASM, we measured DNA methylation of each allele individually and compared them between two alleles. Thus, allele-specific polymerase chain reactions (PCRs) were performed, and given two allele-specific amplicons, we separately measured methylation levels of each allele using pyrosequencing. The allele-specific PCR primers were designed such that each primer of the 3′ end was a pair of bases of heterozygous SNPs (Fig 4).

Bisulfite conversion was performed using the EpiTect Bisulfite Kits (QIAGEN), according to the manufacturer’s instructions. By bisulfite conversion, rs362210701 (C/T) would be converted to (T/T); it was impossible to distinguish the converted T and original T (Fig 4A). Thus, we designed a PCR primer for rs13239907 (A/G), which was located 297 bp downstream of rs36221701 and was in complete linkage disequilibrium with rs36221701 (A→C, G→T: r^2^ = 1.0) in JPT data from the 1,000 genome project [34] (Fig 4B). Allele-specific PCRs were performed, and the two given allele-specific amplicons were analyzed separately using pyrosequencing. Sequence primer 1 targeted rs13239907 was used to check the accuracy of the allele-specific PCR, whereas sequence primer 2 targeted CpG1 and CpG2 were used to analyze the ratio of methylation around rs36221701 (Fig 4C). The primers were designed using the PyroMark Assay Design 2.0 (QIAGEN), supplied by Sigma Aldrich (St Louis, USA). The details of primers are summarized in Fig 4D. PCRs were performed using the PyroMark PCR Kit (QIAGEN), according to the manufacturer’s instructions and by annealing at 54°C. Pyrosequencing was performed using the PyroMark Q24 platform, and initial data analysis was performed using PyroMark Q24 software (Version 2.0.6.20, QIAGEN). We analyzed the ratio of methylation CpG1 and CpG2 between C and T alleles in four heterozygosis samples using a paired *t*-test. The analysis by adding three homozygosis samples of T/T and one of C/C was performed by Wilcoxon signed-rank test.

### Gene expression analysis

Total RNA samples extracted from Tem in the intestinal tissues of 11 IBD patients (seven CD and four UC patients) were used in transcriptome analysis. Library preparation and sequencing were performed by the following procedure. We assessed the quality and quantity of total RNA by analysis of rRNA band integrity on an Agilent RNA 6000 Chip (Agilent Technologies, Santa Clara, USA). cDNA was synthesized by the SMART-Seq v4 Ultra Low Input RNA Kit (Takara Bio, Kusatsu, Japan). We used 500 pg of total RNA to switch template to enrich for full-length cDNA containing the 5′ end of the mRNA and directly add defined PCR adapters to both ends of the first-strand of cDNA. These amplified cDNA were validated using the Agilent 2100 BioAnalyzer High Sensitivity DNA Chip (Agilent Technologies) and the fragments were between 400 and 10,000 bp, yielding approximately 2–10 ng of cDNA. The full-length cDNA was processed with the Low Input Library Prep Kit (Takara Bio). Prior to generating the final library, the amplified cDNA samples were sheared by acoustic Covaris S2 instrument (Covaris, Woburn, USA). Fragments of 200–500 bp were ligated to Illumina’s adapters and PCR amplified. Libraries were quantified using the Agilent 2100 Bioanalyzer (Agilent Technologies) and KAPA Library Quantification Kit (Kapa Biosystems, Woburn, USA). The resulting purified libraries were applied to an Illumina flow cell for cluster generation and sequenced using 100 bp paired-end reads on Hiseq2500 (Illumina, San Diego, USA) sequencer, following the manufacturer's protocols.

Transcriptome analysis was performed using the following procedure. Adapter and low-quality sequences were removed by cutadapt (v1.2.1) [35]. After quality control, poly-A/T sequences were also removed by PRINSEQ (v0.19.2) [36]. For gene expression analysis, the trimmed reads were aligned to the reference human genome (GRCh37/hg19) using TopHat (v2.0.13) [37]. Mapped reads were assembled by Cufflinks (v2.2.1) [38], and the transcripts across all samples were merged by Cuffmerge, a part of the Cufflinks package. The “fragments per kilo base per million map reads” (FPKM) was calculated with Cuffquant.

The analysis of the association between the genotype of rs36221701 and expressions of the genes within 500 kbp up- or downstream of rs36221701 was performed by simple linear regression analysis.

## Results

### ASM around IBD susceptibility genes

Almost 5% (34,154 probes) of the probes on the Japonica array have sites cut by MSREs. A total of 23,371 probes were extracted with two exclusion criteria. Among these, six SNPs were the IBD susceptibility tag-SNPs reported previously [1, 5, 32]. However, only one of the six SNPs showed $\bar{\Delta RAS}$ >0.1, and this result was derived from only one heterozygous sample. Thus, we could not evaluate the ASM of IBD susceptibility tag-SNPs. Most of these tag-SNPs were representative of the associated loci and haplotypes; therefore, we analyzed ASM of SNPs located around IBD susceptibility genes.

Among the 23,371 SNPs, 630 showed $\bar{\Delta RAS}$ >0.1. Twenty-nine SNPs were located within 100 kbp up- or downstream of 200 IBD susceptibility loci [1, 5, 32]. Finally, six SNPs were extracted, excluding inappropriate probes, by visual observation of the signal cluster plots (Fig 2). The six candidate SNPs are summarized in Table 2. The minor allele frequencies in Japanese are referred to 3.5KJPN data by the Tohoku Medical Megabank Project (https://ijgvd.megabank.tohoku.ac.jp/) [34, 39, 40]. The regulatory functions are referred to ENCODE [41], The Sequence Manipulation Suite [42] and The Ensembl Regulatory Build [43].

**Table 2:** Six of candidate *cis*-regulating allele-specific DNA methylation SNPs around inflammatory bowel disease susceptibility genes.

SNP	Chr	Position*	Allele (ref/alt)	MAF in Japanese**	Number of heterozygosity	$\bar{\Delta RAS}$	IBD susceptibility gene [1, 5, 32]	Regulatory function
rs11758967	6	167161726	T/C	0.1213	8	0.11	*RPS6KA2* (intron)	Histone methylation(a)
rs1130368	6	32632818	T/G	0.0135	4	0.23	*HLADQB1* (exon)	CpG island (b)
rs28545822	9	139217805	T/C	0.1737	6	0.18	*CARD9* (40 kbp downstream), *SDCCAG3* (79 kbp upstream), *PMPCA* (87 kbp upstream)	Histone methylation(a)
rs36221701	15	67356489	T/C	0.2888	6	0.14	*SMAD3* (1.7 kbp upstream)	Histone methylation(a) Open chromatin(a) Polymerase 2(a) Promoter Flanking Region(c) Transcription factors(a) (HEY1, BAF155, FOXA1, TAF1, HDAC2)
rs12709500	17	26134974	T/C	0.3435	8	0.16	*NOS2* (exon)	Histone methylation(a) Transcription factor (TAF1) (a)
rs6031593	20	43051213	T/C	0.3422	6	0.12	*HNF4A* (intron)	Histone methylation(a) Open chromatin(a) (c)

*Positions are based on the Genome Reference Consortium human build 37 (GRCh37)

** From 3.5KJPN data by the Tohoku Medical Megabank Project (https://ijgvd.megabank.tohoku.ac.jp/)

(a) ENCODE

(b) CpG Islands

(c) The ensembl regulatory build.

SNP, single-nucleotide polymorphisms; Chr, chromosome; MAF, minor allele frequencies; RAS, relative allele score; IBD, inflammatory bowel disease; RPS6KA2, ribosomal protein S6 kinase A2; HLADQB1, major histocompatibility complex, class II, DQ Beta 1; CARD9, caspase recruitment domain family member 9; SDCCAG3, serologically defined colon cancer antigen 3; PMPCA, peptidase (mitochondrial processing) alpha; SMAD3, SMAD family member 3; NOS2, nitric oxide synthase 2; HNF4A, hepatocyte nuclear factor 4 alpha.

**Fig 5:**
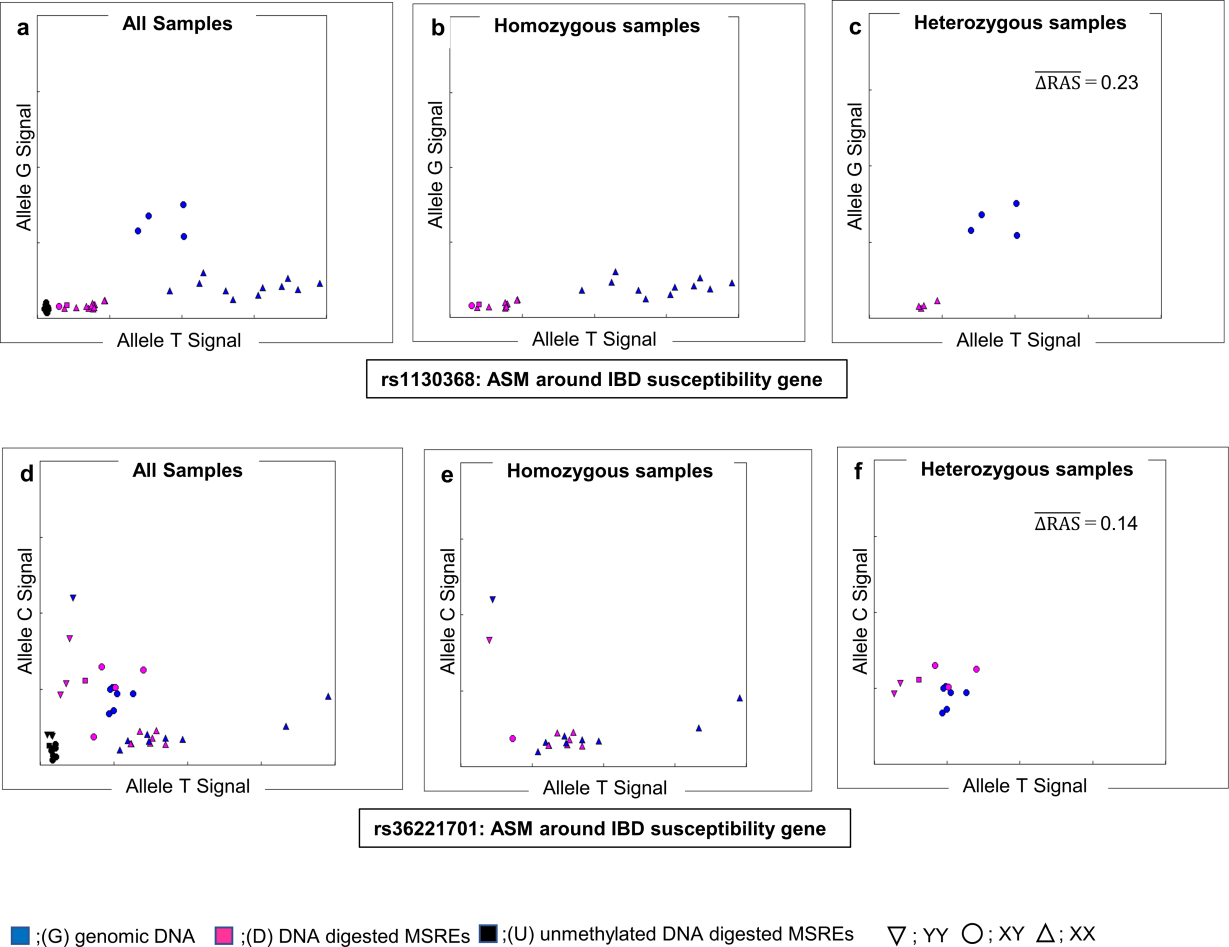
The signal cluster plot in typical allele-specific DNA methylation (ASM) by methylation-sensitive SNP array (MSNP) analysis. (a, b, c) Array data of rs1130368 located around inflammatory bowel disease (IBD) susceptibility gene showing ASM. Rs1130368 is located on the exon of *HLA-DQB1* (6p21. 32). (a) All samples, (b) Homozygous samples, (c) Heterozygous samples. Heterozygous changed to homozygous after digestion ($\bar{\Delta RAS}$ = 0.23 was the top score in candidates). This change was monoallelic (G/T changed only T/T); thus, ASM regulated in *cis* with genotype and not genomic imprinting. This indicated that hypermethylation existed around T allele, whereas hypomethylation existed around G allele. (d, e, f) Array data of rs36221701 located around IBD susceptibility gene showing ASM. Rs36221701 is located on the upstream of *SMAD3* (15q22.33). (d) All samples, (e) Homozygous samples, (f) Heterozygous samples. Heterozygous samples tended to change to homozygous after digestion ($\bar{\Delta RAS}$ = 0.14). This change was monoallelic (C/T changed only C/C); thus, ASM regulated in *cis* with genotype and not genomic imprinting. This indicated that hypermethylation existed around C allele, whereas hypomethylation existed around T allele. RAS, relative allele score.

The top of $\bar{\Delta RAS}$ >0.1 among the candidates was rs1130368 (0.23). This SNP is located on the exon of major histocompatibility complex, class II, DQ Beta 1 (*HLA-DQB1*) (6p21, 32), which is one of the UC susceptibility genes in the European population [1] and one of the CD susceptibility genes in the Japanese population [44]. This SNP showed a typical pattern of ASM (Fig 5A, 5B and 5C). Signals of heterozygosity (G/T) changed to homozygosity (T/T) after digestion. These results indicated that only the G allele was cut by MSREs; in particular, hypermethylated CpG existed around the T allele, whereas hypomethylated CpG existed around the G allele. This change of heterozygosity to homozygosity was monoallelic (G/T changed only to T/T); thus, ASM was regulated in *cis* by genotype, and not genomic imprinting.

Rs36221701 ($\bar{\Delta RAS}$ = 0.14) is located upstream of SMAD family member 3 (*SMAD3*), an IBD susceptibility gene in the European population [1]. This SNP also showed a relatively typical ASM pattern (Fig 5D, 5E and 5F).

### Validate ASM detected by MSNP using bisulfite pyrosequencing

To validate the ASM of Japonica array data, four heterozygous samples of rs36221701 were analyzed using bisulfite pyrosequencing. First, we confirmed the accuracy of allele-specific PCR by pyrosequencing of rs13239907. The product of C allele-specific PCR showed over 98% specificity, and the T allele showed over 86%. Second, we individually analyzed two allele-specific amplicons using pyrosequencing with sequence primer 2 and methylation ratio of two CpGs (Table 3). These results did not reach statistical significance for either CpG1 or CpG2 but indicated a tendency toward hypermethylation around the C allele, rather than T allele (Table 4). Furthermore, by adding homozygosis samples of three T/T and one C/C, significant hypermethylation around the C allele than T allele in both CpG1 (p = 0.022) and CpG2 (p = 0.018) was observed (Table 5). These results were consistent with the results of MSNP analysis using the Japonica array.

**Table 3:** CpGs methylation levels around rs36221701 of each sample by bisulfite pyrosequencing.

Sample	Genotype of rs36221701	CpG1		CpG2	
		C allele (%)	T allele (%)	C allele (%)	T allele (%)
CD4	CT	77	49	67	42
CD5	CT	89	73	86	64
UC1	CT	77	77	74	69
UC4	CT	87	78	82	74
CD7	CC	82	-	76	-
CD6	TT	-	60	-	53
CD8	TT	-	69	-	66
UC5	TT	-	61	-	55

CD, Crohn’s disease; UC, ulcerative colitis.

**Table 4:** Analysis of CpGs methylation levels around rs36221701 comparing with C and T allele in heterozygous samples.

	C allele (n = 4)		T allele (n = 4)		
	Mean	SD	Mean	SD	*p* value*
CpG1 methylation	82.5%	6.4	69.3%	13.7	0.111
CpG2 methylation	77.3%	8.5	62.3%	14.1	0.057

*paired *t*-test.

**Table 5:** Analysis of CpGs methylation levels around rs36221701 comparing with C and T allele in heterozygous samples and adding homozygous samples.

	C allele (n = 5)		T allele (n = 7)		
	Mean	SD	Mean	SD	*p* value**
CpG1 methylation	82.4%	4.0	66.7%	3.4	**0.013**
CpG2 methylation	77.0%	4.3	60.4%	3.7	**0.015**

**Wilcoxon signed-rank test.

### The relation between genotype of rs36221701 and expression of neighboring genes

**Fig 6:**
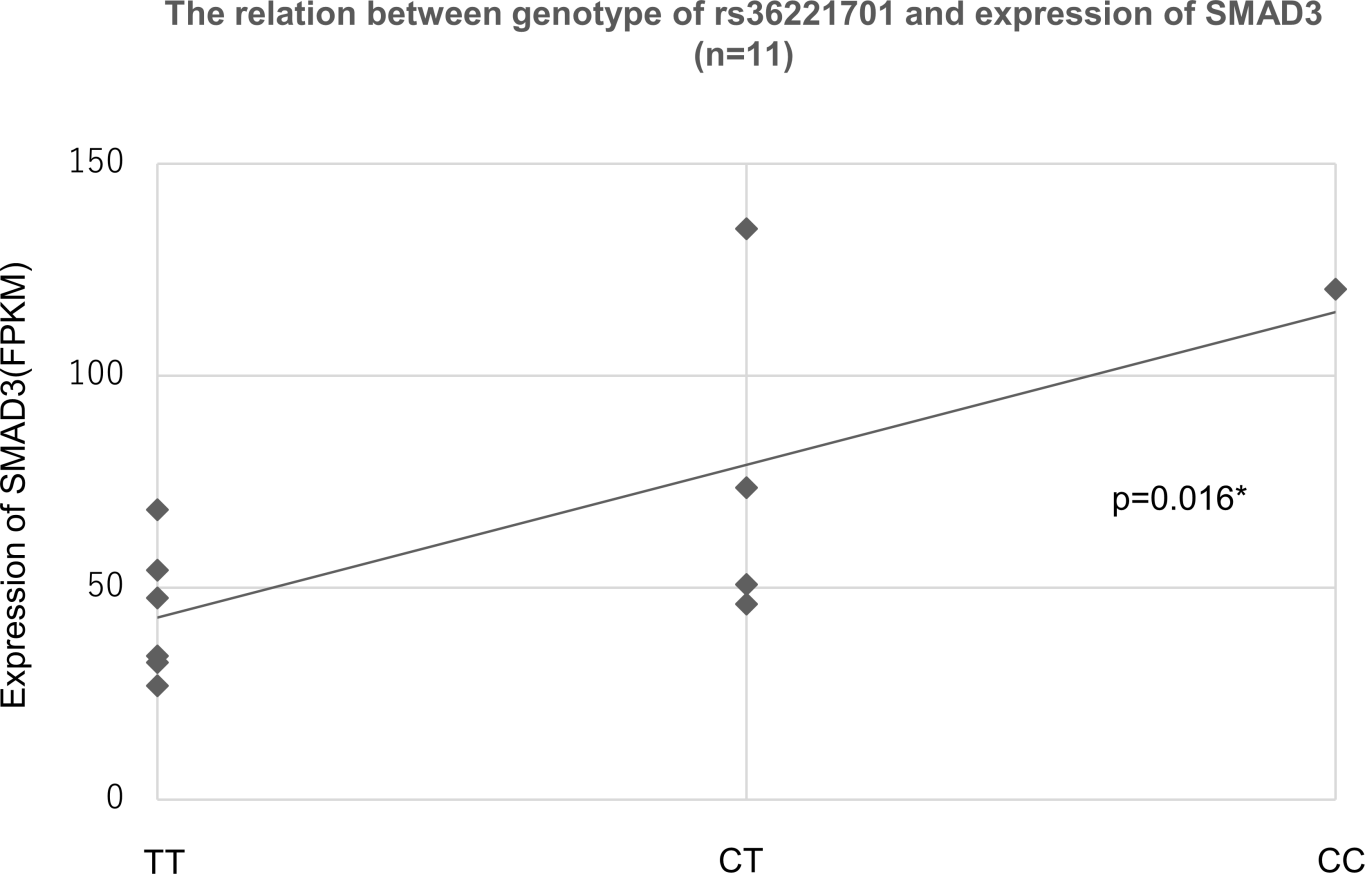
The relation between rs36221701 genotype and SMAD3 expression. The scatter plot shows the expression levels of the genotypes.*Simple linear regression analysis.

Transcriptome analyses were performed for Tem isolated from LPMCs in 11 IBD patients. Six genes existed within 500 kbp up- or downstream of rs36221701; only SMAD3 expression was significantly associated with the genotype of rs36221701 (Table 6, Fig 6). The expression of SMAD3 was enhanced in genotype C/C.

**Table 6:** The relation between genotype of rs36221701 and the expression levels of genes around rs36221701.

Gene	Location	Average expression levels (FPKM)			*p* values*	R^2^	Adjusted R^2^
		TT (n = 6)	CT (n = 4)	CC (n = 1)			
*SMAD6*	15:66994673–67074338	0.02	0	0	-	-	-
*SMAD3*	15:67357990–67487569	43.91	76.33	120.47	**0.0159**	0.494	0.438
*IQCH*	15:67547137–67819641	0.10	0.07	0	-	-	-
*C15orf61*	15:67547137–67819641	17.97	20.62	18.43	0.5012	0.052	-0.054
*MAP2K5*	15:67835020–68099455	10.44	8.42	6.71	0.6636	0.022	-0.087
*SKOR1*	15:68112041–68126174	0.02	0.01	0	-	-	-

*Simple linear regression analysis.

FPKM, fragments per kilobase of exon per million reads mapped.

*SMAD6*, SMAD family member 6; *SMAD3*, SMAD family member 3; *IQCH*, IQ motif containing H; *C15orf61*, chromosome 15 open reading frame 61; *MAP2K5*, mitogen-activated protein kinase kinase 5*; SKOR1*, SKI family transcriptional corepressor 1.

## Discussion

This study is the first report of ASM analysis in IBD, and our results confirmed *cis*-regulated ASM around the IBD susceptibility genes in Tem. In addition, we demonstrated the association between ASM SNP and the expression of SMAD3. These findings have important implications for genetic–epigenetic interactions in IBD patients and provide a mechanism by which SNP susceptibility may exert an effect on susceptibility gene function.

Previous EWAS with IBD reported different results of hyper- or hypomethylation regions; therefore, the methylome of IBD was not confirmed. One of the reasons could be that the samples they analyzed were obtained from peripheral blood [18, 19] or mucosal biopsy tissue [17], which comprised heterogeneous collections of different cell types with very different DNA methylation profiles. Observed DNA methylation profiles were mixtures of the profiles of many types of cells, wherein profiles of heterogeneous tissues were sensitive to tissue cell composition. Analyzing the samples from peripheral blood or mucosal biopsy were appropriate approaches for investigating biomarkers. However, to understand the pathogenesis of IBD, the samples should include disease-relevant cells and consist of relatively homogeneous cells [25, 26]. Ventham et al. [45] reported EWAS, which was performed using immunomagnetically-separated leukocytes in peripheral blood (CD4+ and CD8+ lymphocytes, CD14+ monocytes) from IBD patients. However, there are few reports of analyzing cell-specific methylation in IBD.

In this study, we performed methylation analysis using Tem in LPMCs isolated form the diseased parts of resected intestines from IBD patients. This is the most cell-specific and tissue-specific analysis so far. We selected Tem for analysis because Tem is strongly associated with IBD pathogenesis. First, lymphopenia ameliorates IBD symptoms such as those observed in patients with active HIV infections [46], or in patients undergoing bone marrow transplantation [47]. Second, both CD and UC are frequently associated with other T-cell mediated diseases (i.e., psoriasis and multiple sclerosis) [48]. Third, colitis can be induced in immunodeficient mice by transferring naïve T cells [49]. Fourth, strategies blocking T-cell function are useful for attenuating mucosal inflammation in mice with experimental colitis [50]. We confirmed cell homogeneity of Tem from intestinal tissues by flow cytometry, and no or lower expressions of CD20 (MS4A1) and CD19 (B cell marker), CD14 (macrophage marker), and CD244 (NK cell marker) from the results of transcriptome analysis further confirmed these findings.

In this study, heterozygous SNPs were used for ASM analysis. In general, to analyze the association between methylation levels and genotypes, methylation levels are compared among three groups: homozygous for the enhanced allele, homozygous for the suppressed allele, and heterozygous. However, it requires numerous samples of each genotype to obtain statistical significance and it is difficult to prepare sufficient samples because IBD is a relatively rare disease in Japan, and few patients undergo surgical resection. Furthermore, DNA methylation is influenced by the type of cell and tissue species, and also environmental factors such as smoking, diet, inflammation, and pharmacotherapy. Usually, it is impossible to establish consistent environmental factors among individuals. To solve this problem, we compared methylation levels between each of the two alleles of heterozygous SNPs in the same individual, thus standardizing the environmental factors and analyzing the *cis*-regulated ASM.

ASM analysis is proliferating and becoming highly relevant as we move into the post-genome sequencing and post-GWAS era [24]. ASM is present in 1.5%–10% of CpG [23, 51–53], although the identified ASMs depend on sample sizes and thresholds. Our microarray data showed 630 ASMs (2.7%) in 23,371 SNPs filtered by our criteria (Fig 1), and our results are comparable to previous reports. The associations of *cis*-regulated ASM with multiple variants implicated in complex phenotypes such as celiac disease, IgA nephropathy, and UC and CD [52] were reported even in the analysis of peripheral blood with healthy control volunteers. We extracted six candidate *cis*-regulating ASM SNPs around IBD susceptibility genes. Among the six candidates, the top of $\bar{\Delta RAS}$ >0.1 was rs1130368 (6p21.32) located on exon of *HLA-DQB1*, which is a UC susceptibility gene in the European population [1] and one of the CD susceptibility genes in the Japanese population [44]. It is difficult to analyze the ASM of HLA region by bisulfite sequencing because the region contains too many SNPs to design the primers. One of the six candidates, rs36221701, is located upstream of *SMAD3*, which is an IBD susceptibility gene in the European population [1] and is implicated in patients who require recurrent surgery with CD [54]. *SMAD3* is a transcriptional modulator activated by transforming growth factor-beta, a famous anti-inflammatory cytokine. We compared the ratio of DNA methylation of two CpGs around rs36221701 between both alleles using bisulfite pyrosequencing. In heterozygous samples, the results did not reach statistical significance in both CpGs because of small number of samples. By adding homozygous samples, significant hypermethylation around C allele and not T allele in both CpGs was observed. These results were consistent with the results of MSNP analysis using the Japonica array.

Transcriptome analysis showed a statistically significant relationship between the genotype of rs36221701 and the expression of *SMAD3*. The results showed that expression of *SMAD3* was enhanced in the hypermethylated genotype. Generally, DNA hypermethylation in the CTCF-binding site or promoter regions results in transcriptional gene repression by inhibiting the binding of CTCF or the enhancer to DNA; however, this regulation is not consistent, and sometimes hypermethylation, particularly in the gene body, results in transcriptional gene enhancement [55, 56]. Many factors besides DNA methylation, such as transcription factor and micro RNA [57], influence gene expression; thus, it is impossible to conclude that the ASM detected regulated the expression by only our results. Our candidate SNPs are not located on known CTCF-binding sites or promoter regions. Previously reported ASMs showed a tendency toward being located outside of CpG island and further away from genes [58, 59]; moreover, these are not often located on known CTCF-binding sites or promoter regions [60]. ASMs located on CTCF-binding sites indicate expression regulation [60]; however, such cases are rare. DNA configures the 3D looping of chromatin into large and complex topologically associating domains (TADs) [61, 62]; thus, ASMs may influence the transcription at regions that are distant (several Mbp or more) from them. As described above, although the detected ASM is not located on CTCF-binding sites or promotor regions, it may possibly influence the regulation of *SMAD3* expression. Namely, regulating the expression of *SMAD3* by DNA methylation could be a mechanism for developing IBD susceptibility. However, the function and significance of ASM are still unclear, and further ASM analyses are needed to understand the mechanisms by which ASM influences gene expression.

The most serious limitation of our study was the requirement for surgical specimens to analyze DNA methylation in the inflammatory intestinal mucosa. It is difficult to prepare sufficient samples because IBD is a relatively rare disease in Japan, and few patients undergo surgical resection. As a result, there were a small number of samples analyzed as IBD without distinction between CD and UC. Moreover, the number of LPMCs that could be isolated from surgical specimens was too few to analyze several immunocompetent cells, such as T cells, B cells, dendritic cells, and macrophages; we could only analyze Tem. The increasing number of samples and cell species of immunocompetent cells may more certainly show ASM, although this is a subject for future analysis.

The other problem inherent to analyzing surgical specimens is that the etiology of severe active IBD that requires surgical resection may be different from the etiology of mild active IBD that does not require surgical resection. Patients with mild active IBD are the majority, and IBD that requires surgical resection may be a specialized form of IBD that has a different etiology. Therefore, we cannot state clearly that our results are generalizable to all cases of IBD. However, the analysis of heterogeneous collections such as mucosal biopsy tissues has limitations, and we think that our study using surgical specimens is very important for confirming the methylome of IBD.

Other limitations included the following: (1) the probes used in this study were only those with sites cut by MSREs, which comprise 5.2% of all probes. (2) All subjects were limited to heterozygous SNPs. (3) The subjects only included patients with severe IBD who underwent surgery; mild IBD patients who did not require surgery were not included, and these include a major proportion of IBD patients. (4) We do not know the appropriate threshold of ASM with the Japonica array. (5) We could not analyze the effect of several environmental factors and medications due to the small sample size. (6) Finally, the influence of collagenase on DNA methylation when isolating LPMCs was not controlled.

In conclusion, we confirmed the existence of *cis*-regulated ASM around IBD susceptibility genes. We also confirmed the association between the genotype of the ASM SNP (rs36221701) and the expression of *SMAD3*, one of the IBD susceptibility genes. These results suggest that DNA methylation mediates genetic effects related to disease susceptibility. However, the exact mediation process of the interaction between genetic and epigenetic mechanism is still unclear, and further analyses will be needed.
