# Correction: Allele-specific DNA methylation of disease susceptibility genes in Japanese patients with inflammatory bowel disease

**DOI:** 10.1371/journal.pone.0212148

**Published:** 2019-02-05

**Authors:** Hirofumi Chiba, Yoichi Kakuta, Yoshitaka Kinouchi, Yosuke Kawai, Kazuhiro Watanabe, Munenori Nagao, Takeo Naito, Motoyuki Onodera, Rintaro Moroi, Masatake Kuroha, Yoshitake Kanazawa, Tomoya Kimura, Hisashi Shiga, Katsuya Endo, Kenichi Negoro, Masao Nagasaki, Michiaki Unno, Tooru Shimosegawa

The affiliation for the 16^th^ author is incorrect. Masao Nagasaki is not affiliated with #2 but with #3 Tohoku Medical Megabank Organization, Tohoku University, Sendai, Japan.
